# Multimodality Thoracoabdominal Imaging Findings in a Rare Case of Thoracic Endometriosis Syndrome

**DOI:** 10.7759/cureus.6819

**Published:** 2020-01-30

**Authors:** Jigarkumar Rangunwala, Juliana Sitta, Kshama Vyas, Manohar Roda

**Affiliations:** 1 Radiology, University of Mississippi Medical Center, Jackson, USA; 2 Radiology, University of Mississippi Medical Center, Jackson, USA; 3 Family Medicine, University of Mississippi Medical Center, Jackson, USA

**Keywords:** catamenial hemothorax, thoracic endometriosis syndrome, advanced endometriosis, pelvic endometriosis, mri, pelvic mri, chest mri

## Abstract

Thoracic endometriosis syndrome (TES) is an extremely rare disorder, and it is defined as the presence of functional endometrial tissue in pleura, airways, and lung parenchyma. We describe a rare case of a 29-year-old nulliparous female who presented with abdominal pain, dyspareunia, and shortness of breath. She complained of worsening of symptoms around the menstrual cycle. Initial workup showed markedly elevated CA-125 levels. A chest radiograph and CT of the chest, abdomen, and pelvis demonstrated large tension hydrothorax, ascites, and bilateral ovarian cysts. A chest tube was placed to decompress the tension hydrothorax, which drained copious amounts of blood. In view of the unexplained etiology of large hemothorax and elevated CA-125 levels, an MRI of the abdomen and pelvis was performed. This revealed advanced pelvic endometriosis, a right pleural nodule, and ipsilateral hydropneumothorax. Based on these findings, a diagnosis of TES was presumed. The patient was then referred to video-assisted thoracoscopy (VATS) and continuous estrogen suppression for optimal treatment. On early follow-up, she presented with recurrent hydropneumothorax, which was successfully managed with CT-guided chest tube placement and remained stable on further follow-ups. TES diagnosis is often challenging and delayed, demanding a high index of suspicion in patients with risk factors and characteristic clinical presentation. Radiologists should be aware of key imaging findings to help in early diagnosis for timely clinical and surgical management.

## Introduction

Endometriosis is defined as the presence of functional endometrial tissue outside of the uterine cavity [1]. It is commonly found in the pelvis and less often in the abdomen, thorax, brain, or skin. Thoracic endometriosis syndrome (TES) is a rare disorder most commonly presenting with catamenial pneumothorax (73%), catamenial hemothorax (14%), hemoptysis (7%), or lung nodules (6%) [2]. There is a strong association between TES and pelvic endometriosis (50-84%) [2]. We report the case of a 29-year old woman with advanced multifocal pelvic endometriosis associated with TES causing catamenial massive hemothorax, recurrent pneumothorax, and a hemorrhagic/cystic pleural nodule.

## Case presentation

A 29 years-old nulliparous African-American female presented to the emergency department with abdominal pain and bloating for a week, which had progressively worsened, without any relief from over-the-counter medications. The patient also developed shortness of breath while lying on her right side, which progressed to both sides. She denied dysmenorrhea, but reported dyspareunia and worsening of symptoms around the menstrual cycle.

**Figure 1:**
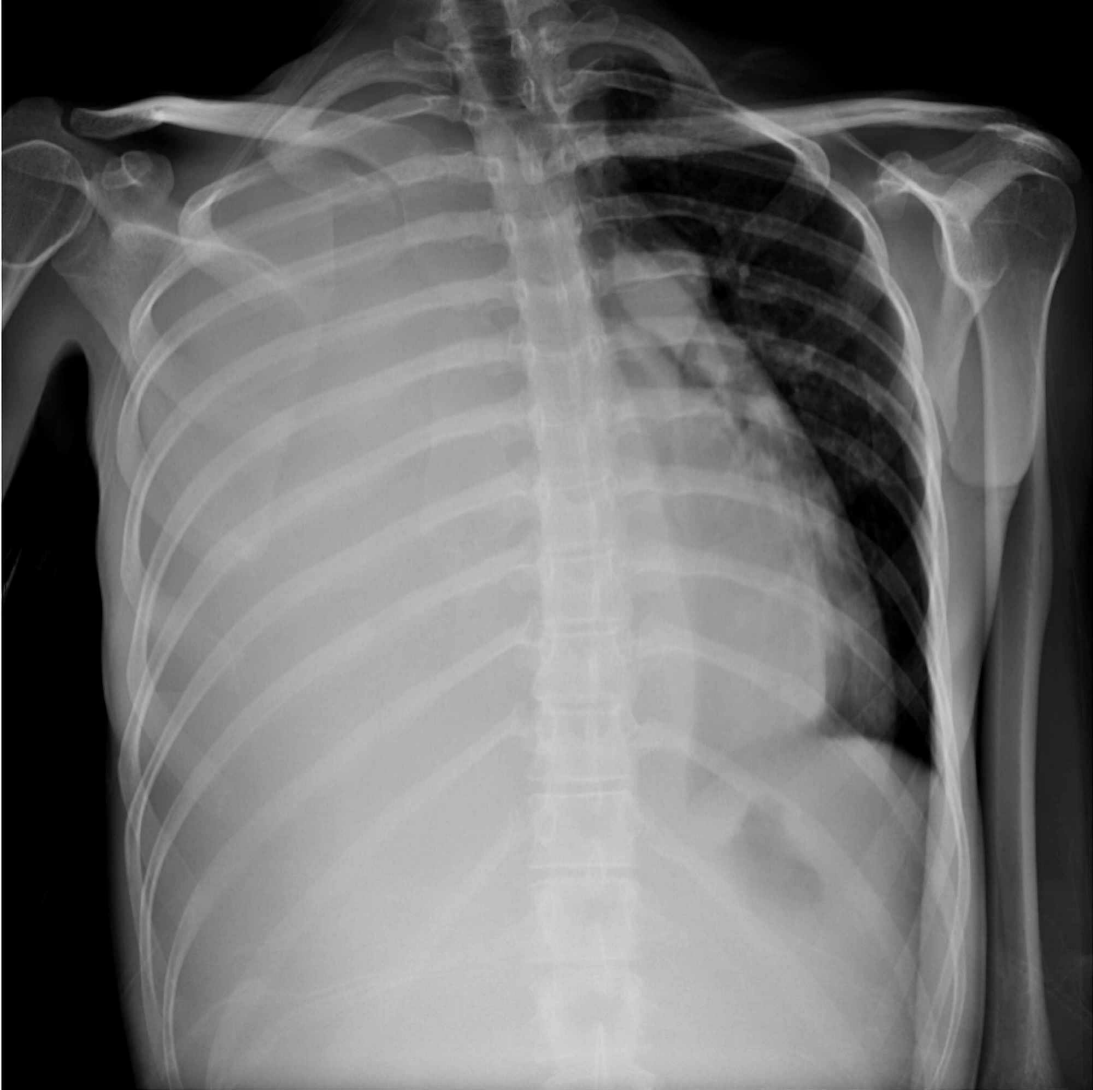
Tension hydrothorax: chest radiograph demonstrates a large right pleural effusion with mediastinal shift to the left; no sign of pneumothorax

**Figure 2:**
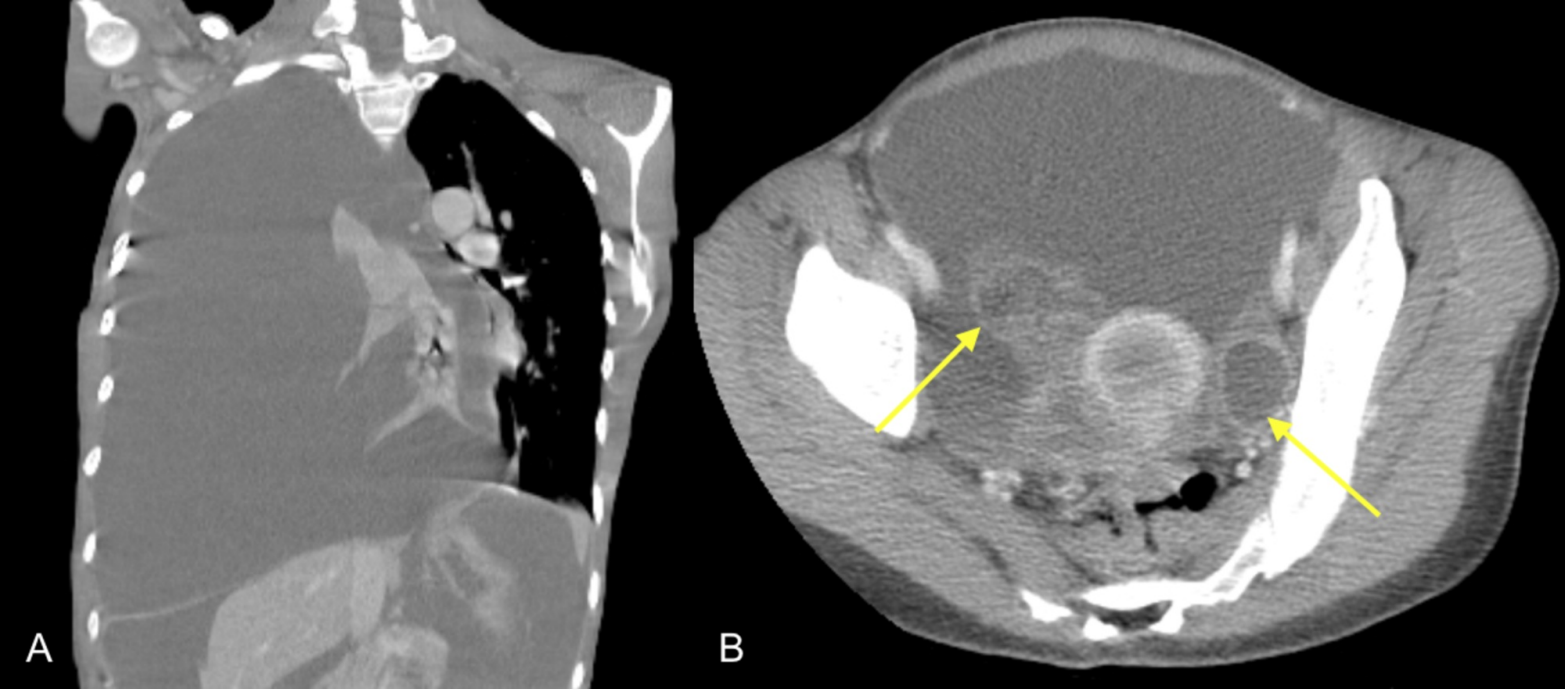
Tension hydrothorax: ascites and ovarian cysts A: coronal CT image of the chest demonstrates a large right-sided pleural effusion with indeterminate density (30 HU), collapse of the right lung, inversion of the right hemidiaphragm, and a leftward shift of the mediastinum, compatible with tension hydrothorax. B: axial CT image of abdomen-pelvis demonstrates ascites with bilateral ovarian cysts CT: computed tomography; HU - Hounsfield units

**Figure 3:**
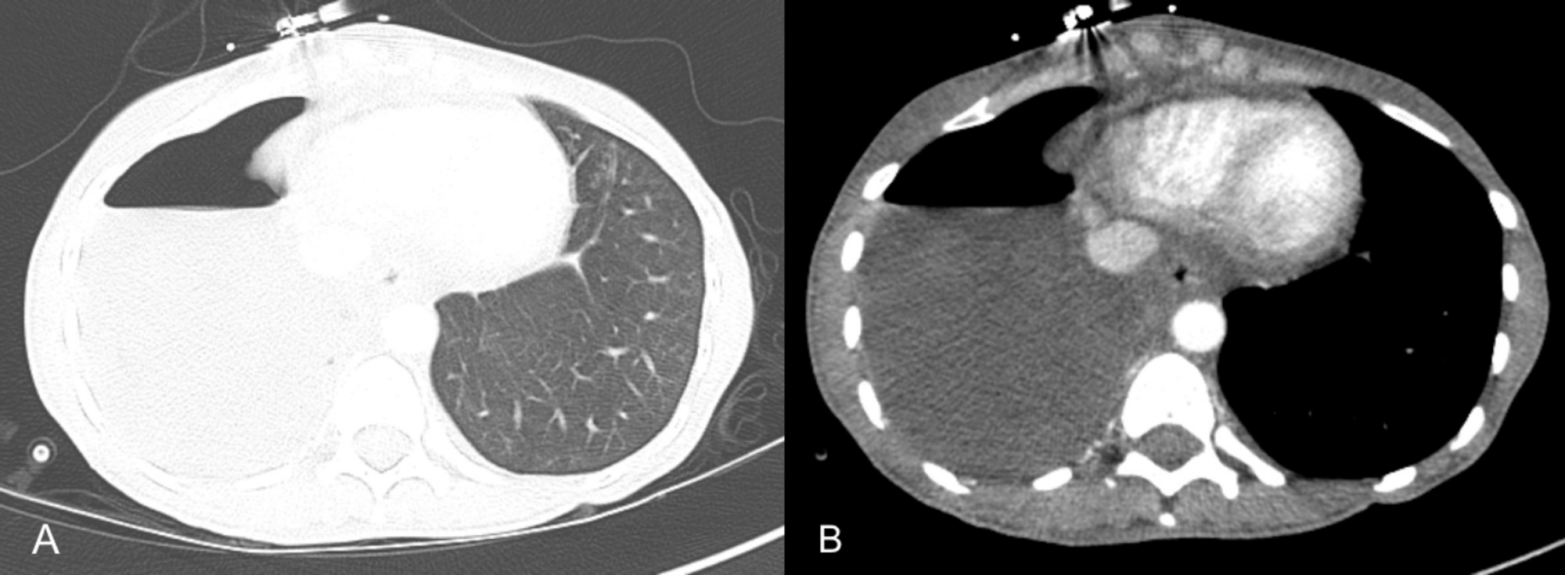
Hydropneumothorax with persistent right pleural effusion Axial CT chest images including (A) lung window and (B) mediastinal window demonstrating right-sided hydropneumothorax with air-fluid level CT: computed tomography

Initial chest radiograph performed during admission demonstrated a large right-sided pleural effusion, a leftward shift of the mediastinum, and inversion of the diaphragm, compatible with tension hydrothorax (Figure 1). This was further evaluated with CT of the chest, abdomen, and pelvis, which confirmed pleural effusion of indeterminate density [mean attenuation of 30 Hounsfield units (HU)] and revealed ascites as well as bilateral ovarian cysts (Figure 2). Serum CA-125 ordered during the initial workup was markedly elevated (80 µ/mL). Cytology studies of the pleural effusion and ascites showed hemorrhagic blood products but did not show any evidence of infection or malignancy. The surgical team was consulted for chest tube placement to drain the right-sided pleural effusion which drained thick dark hemorrhagic fluid. Repeat CT of the chest three days after chest tube placement revealed pneumothorax with persistent right pleural effusion (Figure 3).

**Figure 4:**
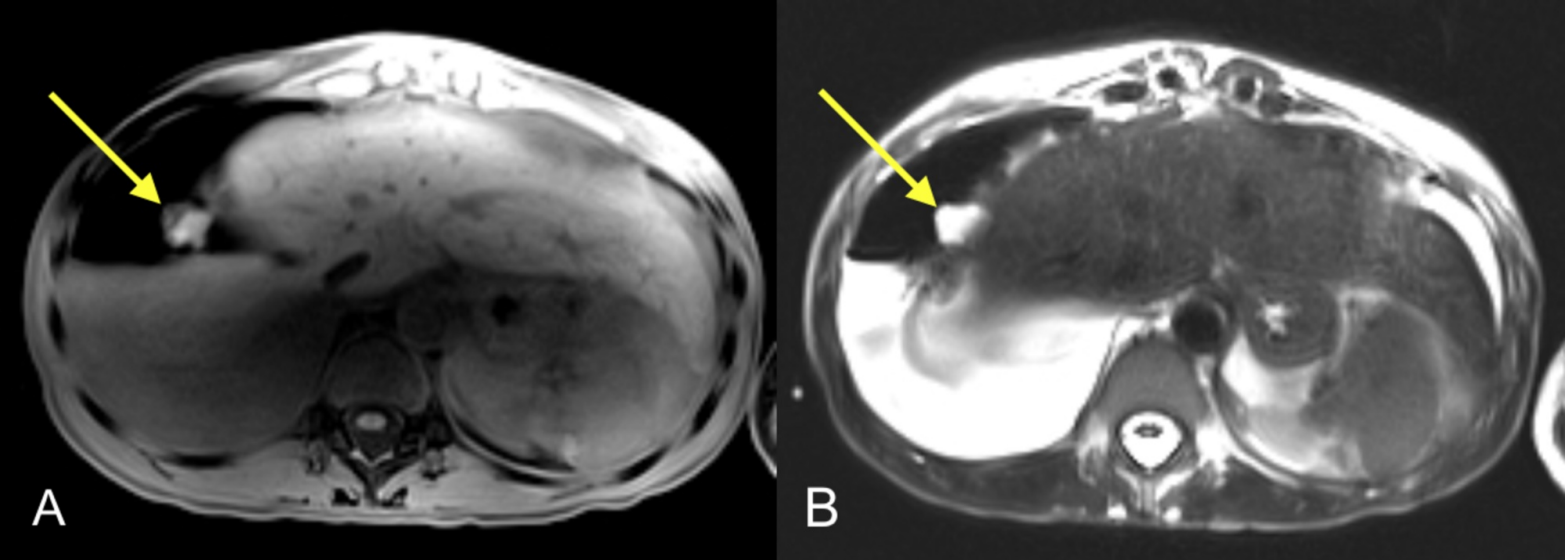
Hemorrhagic right pleural nodule MRI images of the chest including (A) axial T1 image and (B) axial T2 image demonstrate heterogeneous soft tissue/fluid signal intensity hemorrhagic right pleural nodule. This nodule is measuring 2 cm (yellow arrows) and shows T1/T2 hyperintense signal without significant enhancement (not shown). Right-sided hydropneumothorax is re-demonstrated MRI: magnetic resonance imaging

**Figure 5:**
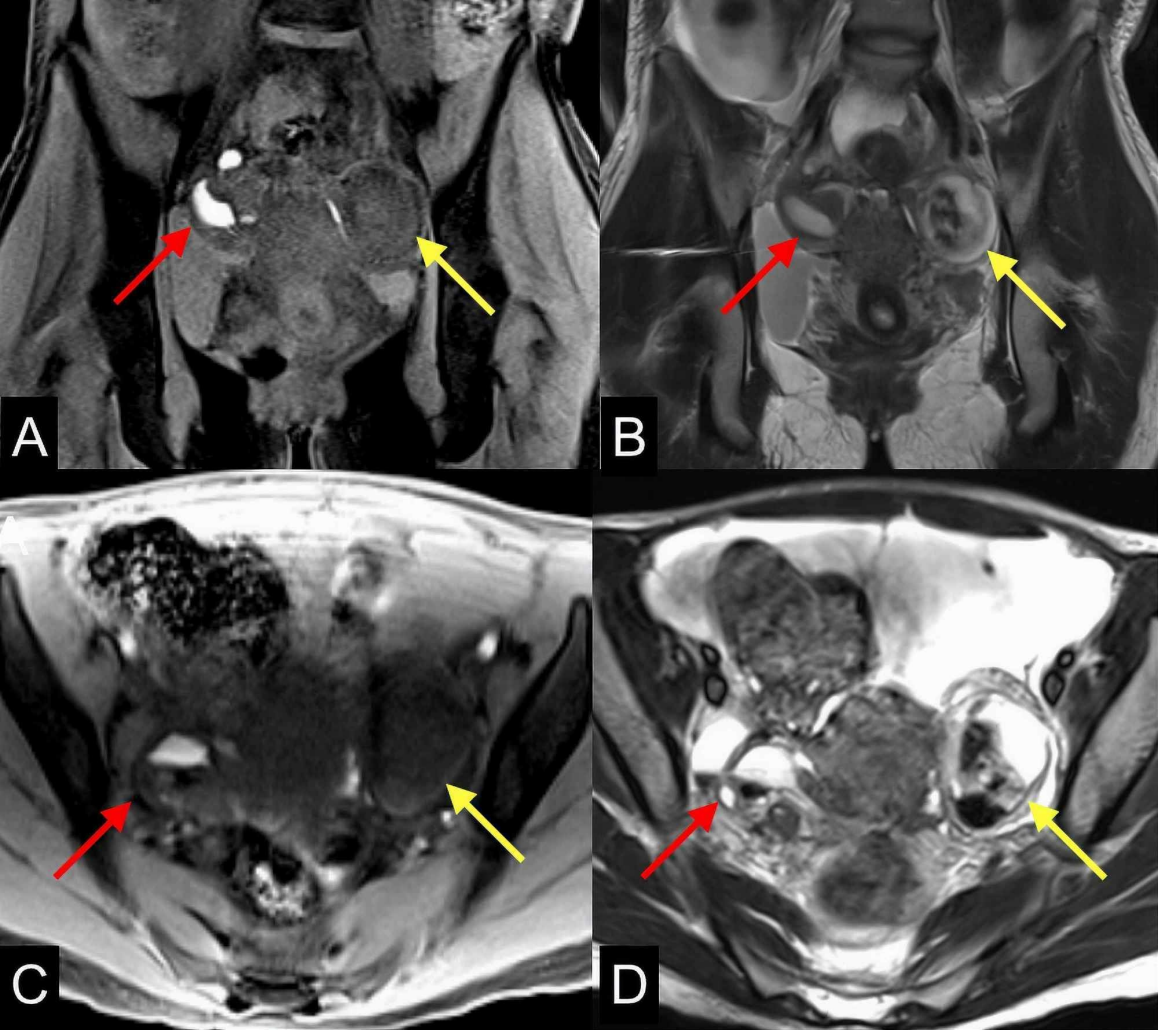
Hemorrhagic adnexal masses Multiple MRI images of the pelvis (A) coronal T1 GRE with fat saturation, (B) coronal T2, (C) axial T1 GRE with fat saturation, and (D) axial T2 demonstrate complex heterogeneous hemorrhagic right (red arrows) and left (yellow arrows) adnexal masses with chronic hydrosalpinx. These bilateral adnexal masses show areas of T1 hypo/hyperintense and T2 hyper/hypointense signal representing hemorrhagic blood products without enhancement (not shown) MRI: magnetic resonance imaging; GRE: gradient recalled echo

**Figure 6:**
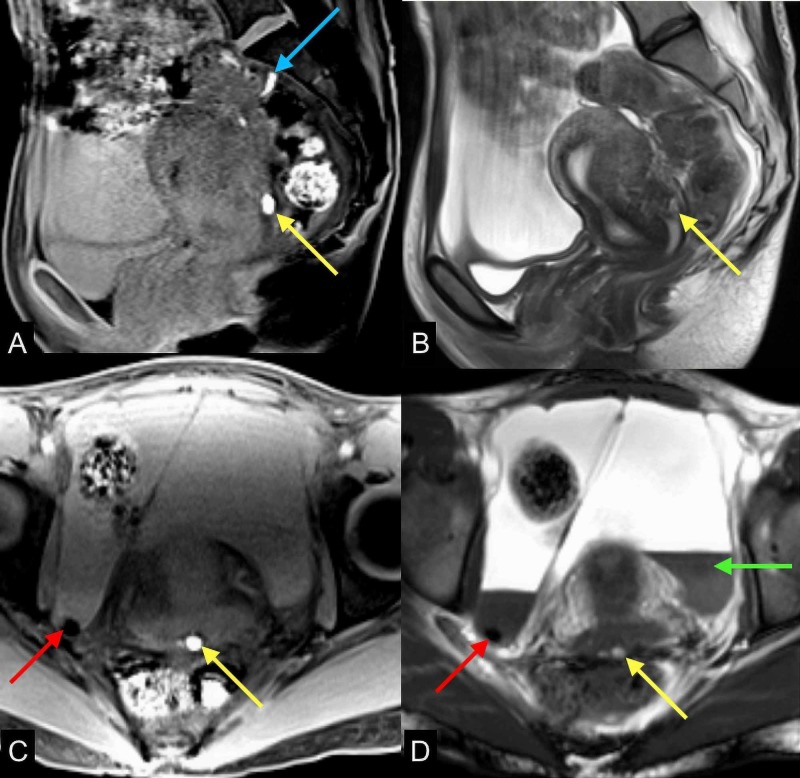
Advanced endometriosis with hemoperitoneum Multiple MRI images of the pelvis (A) sagittal T1 GRE with fat saturation, (B) sagittal T2, (C) axial T1 GRE with fat saturation, and (D) T1 GRE demonstrate nodular peritoneal thickening with focal T1/T2 hypointense nodules (red arrows) in the right hemipelvis associated with blooming, representing hemosiderin deposits related to endometriomas. There are also T1 hyperintense, andT2 hypointense multiple hemorrhagic implants along the uterine serosal margin (yellow arrows) and rectal serosal margin (blue arrow). Complex ascites with fluid-fluid levels due to dependent layering of high protein/hemorrhagic contents show T1/T2 hypo/hyperintense signal (green arrow) MRI: magnetic resonance imaging

MRI of the abdomen and pelvis was recommended for further evaluation in view of elevated levels of serum CA-125, unexplained etiology of massive right hemorrhagic pleural effusion, and ovarian cysts. MRI demonstrated a right pleural hemorrhagic/cystic non-enhancing 2-cm nodule (Figure 4), moderate ascites, multifocal hemorrhagic nodular lesions in the aortocaval region, along pelvic sidewalls and adnexal regions, as well as pouch of Douglas characteristic of multiple endometriomas. Pelvic MRI characterized adnexal abnormalities as hemorrhagic bilateral tubo-ovarian masses, associated hydrosalpinx, with internal dependent fluid-fluid levels and thin septations. Also, there was generalized and nodular peritoneal thickening, hemorrhagic implants along the posterior lower abdominal wall, and uterine serosal margins. Overall, the MRI findings indicated advanced pelvic endometriosis (Figures 5 and 6).

Based on the spectrum of these multiple chest-abdominal-pelvic findings associated with recurrent hemorrhagic right pleural effusion and a pleural hemorrhagic/cystic nodule, a diagnosis of TES was suggested. The patient underwent video-assisted thoracoscopy (VATS), with wash-out of the hemorrhagic pleural effusion, pleurodesis, and chest tube placement. No solid nodules or masses were appreciated on VATS. The chest tubes were subsequently removed with uneventful postoperative recovery. The patient received Lupron 11.25 mg intramuscular (IM) injection (AbbVie Inc., Chicago, IL) for ovarian suppression upon discharge and followed up with gynecology and surgical specialties.

**Figure 7:**
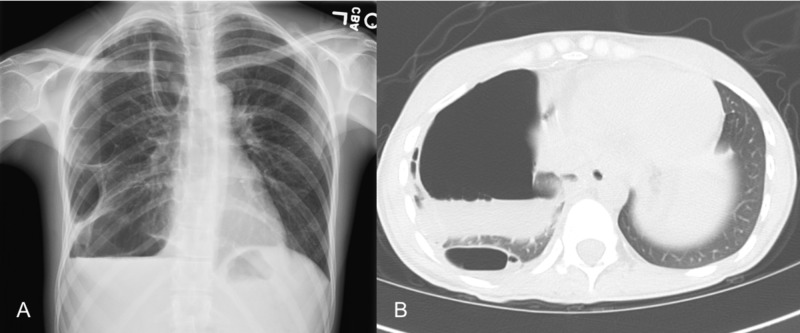
Recurrent hydropneumothorax Follow-up chest radiograph (A) and axial CT chest image (B) demonstrate right-sided recurrent progressive multi-loculated hydropneumothorax CT: computed tomography

**Figure 8:**
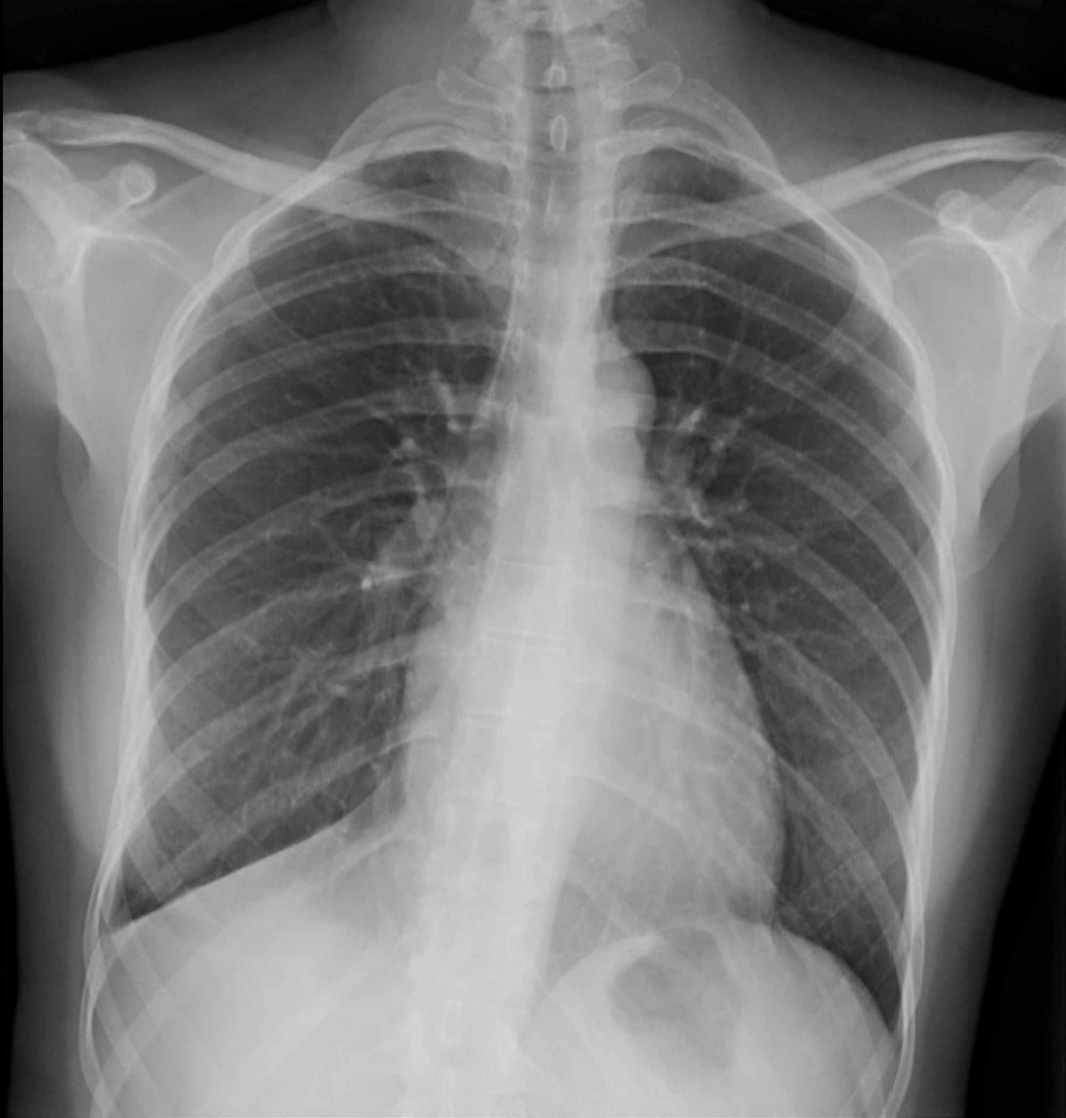
Resolution of the condition Follow-up normal chest radiograph that shows complete resolution of the hydropneumothorax

The patient was readmitted 10 days after discharge, in view of recurrent multi-loculated right-sided hydropneumothorax (Figure 7). A CT-guided chest tube placement was again performed with an unremarkable postoperative course and subsequent resolution of the hydropneumothorax. The patient followed up with gynecology as scheduled every three months for Lupron 11.25 mg IM injection for a total of five injections and remained stable. She was then transitioned to combined oral contraceptive pills indefinitely. The patient continues to have regular six month-yearly follow-ups and remains stable (Figure 8). 

## Discussion

Endometriosis is a common disorder, affecting 6-10% of women in reproductive age [3]. It is defined as the presence of ectopic functional endometrial tissue, most commonly found in the ovaries, pelvic peritoneum and, less commonly, in C-section scars, deep subperitoneal tissues, gastrointestinal tract, bladder, chest, and subcutaneous tissue. Patients can be either asymptomatic, or they can present with menstrual pelvic pain, non-menstrual pelvic pain, infertility, and dyspareunia [1].

TES is a rare disorder, and it is defined as the presence of functional endometrial tissue in pleura, lung parenchyma, or tracheobronchial system [4]. TES affects the right hemithorax in 92% of the cases (most commonly the pleural surface and less frequently the lung parenchyma), with a mean age at presentation of 35 ±0.6 years (range: 15-54) [2,5].

There are different mechanisms suggested for the pathogenesis of TES, such as the trans-diaphragmatic migration, which comprises the following: 1) tissue migration through pelvic vessels; 2) reflux of endometrial tissue through fallopian tubes into the peritoneal cavity; and 3) progression into the thoracic cavity through diaphragmatic fenestrations/defects. This mechanism explains the right-side asymmetry in the distribution of TES, believed to occur due to the pooling of leaked endometrial cells by the falciform ligament. Metaplasia of coelomic epithelium is also a discussed mechanism for the pleural type thoracic endometriosis [6].

Symptomatic patients with TES usually present with symptoms of chest pain, followed by dyspnea, hemoptysis, and cough (rare) within 24-48 hours of the onset of menses [2]. Diagnostic imaging methods include chest X-ray, CT, and MRI. VATS remains the gold standard for diagnosis and is also the treatment of choice, as it allows for procedures such as the closure of diaphragmatic defects, mechanical pleurodesis, chemical pleurodesis, and lung resection. The value of cytologic studies of pleural fluid remains uncertain. Although of low specificity, serum levels of CA-125 can be an indicator of serosal abnormalities (with a cut-off level of 39 µ/mL), as there is no specific biomarker available for endometriosis currently [7,8]. 

In the initial assessment of patients presenting with chest symptoms, radiographic and chest CT scan studies usually demonstrate pleural effusion, pneumothorax, ground-glass infiltrates, thin wall cavities, nodules, and bullous formation. Abdomen and dedicated pelvic MRI can corroborate the diagnosis of endometriosis, as pelvic endometriosis is highly associated with TES [2,7,8].

MRI is currently the preferred imaging method to assess endometriosis with high sensitivity and specificity [1,9]. Its advantages include the possibility of a complete assessment of abdominal compartments, the ability to map disease extension, identify anatomical relations and variants, assess associated complications, pre-surgical planning, and post-surgical follow-up without the use of ionizing radiation [1,10]. Fat- saturated T1-weighted sequence has contributed to the MRI accuracy for endometriosis diagnosis, improving lesion depicting capacity and differentiation between blood and fat-containing lesions [9]. Since surgical excision of endometriotic lesions remains the treatment of choice, accurate pre-surgical description of foci is crucial for better outcomes [10].

Endometriosis can have different presentations on MRI, depending on the location, degree of fibrosis, and the condition of hemorrhage (acute or chronic). Fibrous and compact smooth muscle tissue present with intermediate signal intensity on T1 images and low signal intensity on T2 images. However, deep pelvic endometriotic foci present as irregular nodules, with a hyperintense signal on T1 images and hypointense signal on T2 images. Pelvic adhesions, tubal blockage, and ovarian endometriomas can result in tubo-ovarian complex masses. The presence of dilated ectopic endometrial glands may result in a blended appearance of high-intensity signal areas on T2 images with a variable signal on T1 images, depending on the stage and degree of hemorrhage [9]. The hemorrhagic cysts are typically characterized by hyperintense signal on T1 images and hypointense signal on T2 images due to deoxyhemoglobin and methemoglobin content [9,11].

Treatment modalities include clinical and/or surgical management. Medical therapy consists of hormonal suppression of ovarian estrogen secretion that can be achieved with oral contraceptives, progesterone agonists, gonadotropin-releasing hormone agonists, and danazol. A combination of medical and surgical management is associated with low recurrence in a long-term follow-up [8,9,12].

## Conclusions

TES is an extremely rare condition. Consequently, its diagnosis is often challenging and delayed, demanding a high index of clinical suspicion in patients with risk factors and characteristic clinical presentation. Radiologists should be aware of the key imaging findings to help guide early and proper clinical and surgical management.
